# Quantifying resistance to very-long-chain fatty acid-inhibiting herbicides in *Amaranthus tuberculatus* using a soilless assay

**DOI:** 10.1371/journal.pone.0295927

**Published:** 2023-12-22

**Authors:** Dylan R. Kerr, Jeanaflor Crystal T. Concepcion, Seth A. Strom, Dean E. Riechers

**Affiliations:** Department of Crop Sciences, University of Illinois, Urbana, IL, United States of America; Washington University, UNITED STATES

## Abstract

Resistance to preemergence (PRE) soil-applied herbicides, such as inhibitors of very-long-chain fatty acid (VLCFA) elongases, was documented in two waterhemp [*Amaranthus tuberculatus* (Moq.) J.D. Sauer] populations (SIR and CHR) from Illinois, USA. To limit the spread of resistant weed populations, rapid detection measures are necessary. Soil-based resistance assays are limited by edaphic factors, application timing, variable seeding depth and rainfall amount. Therefore, cost-effective techniques mitigating effects of edaphic factors that are appropriate for small- to large-scale assays are needed. Our research goal was to identify and quantify resistance to the VLCFA-inhibiting herbicides, *S*-metolachlor and pyroxasulfone, using a soilless greenhouse assay. Dose-response experiments were conducted under greenhouse conditions with pre-germinated waterhemp seeds planted on the vermiculite surface, which had been saturated with *S*-metolachlor (0.015–15 μM), pyroxasulfone (0.0005–1.5 μM), or *S*-metolachlor plus the cytochrome P450 (P450) inhibitor, malathion. Lethal dose estimates of 50% (LD_50_) and growth reduction of 50% (GR_50_) were calculated for *S*-metolachlor and pyroxasulfone PRE and used to determine resistance indices (RI) for resistant populations (CHR and SIR) relative to sensitive populations, SEN and ACR. RI values for *S*-metolachlor using LD_50_ values calculated relative to SEN and ACR were 17.2 and 15.2 (CHR) or 11.5 and 10.1 (SIR), while RI values for pyroxasulfone using LD_50_ values calculated relative to SEN and ACR were 3.8 and 3.1 (CHR) or 4.8 and 3.8 (SIR). Malathion decreased the GR_50_ of *S*-metolachlor to a greater degree in CHR compared to ACR, consistent with P450 involvement in *S*-metolachlor resistance in CHR. Results from these soilless assays are in accord with previous findings in soil-based systems that demonstrate CHR and SIR are resistant to *S*-metolachlor and pyroxasulfone. This method provides an effective, reproducible alternative to soil-based systems for studying suspected PRE herbicide-resistant populations and will potentially assist in identifying non-target-site resistance mechanisms.

## Introduction

Preemergence (PRE) soil-applied herbicides are an integral resource for residual weed control [1–4]. Very-long-chain fatty acid (VLCFA)-inhibiting herbicides are effective for selective PRE control of annual grasses and small-seeded broadleaf weed species in corn (*Zea mays* L.) and soybeans [*Glycine max* (L.) Merr] [5,6]. Since their development in the 1950s, an increased use of VLCFA-inhibiting herbicides has resulted from reduced tillage practices [7], the growing demand for soil-residual herbicides due to limited options for broadleaf weed control [8,9], and integrated pest management strategies [10,11].

**Fig 1:**
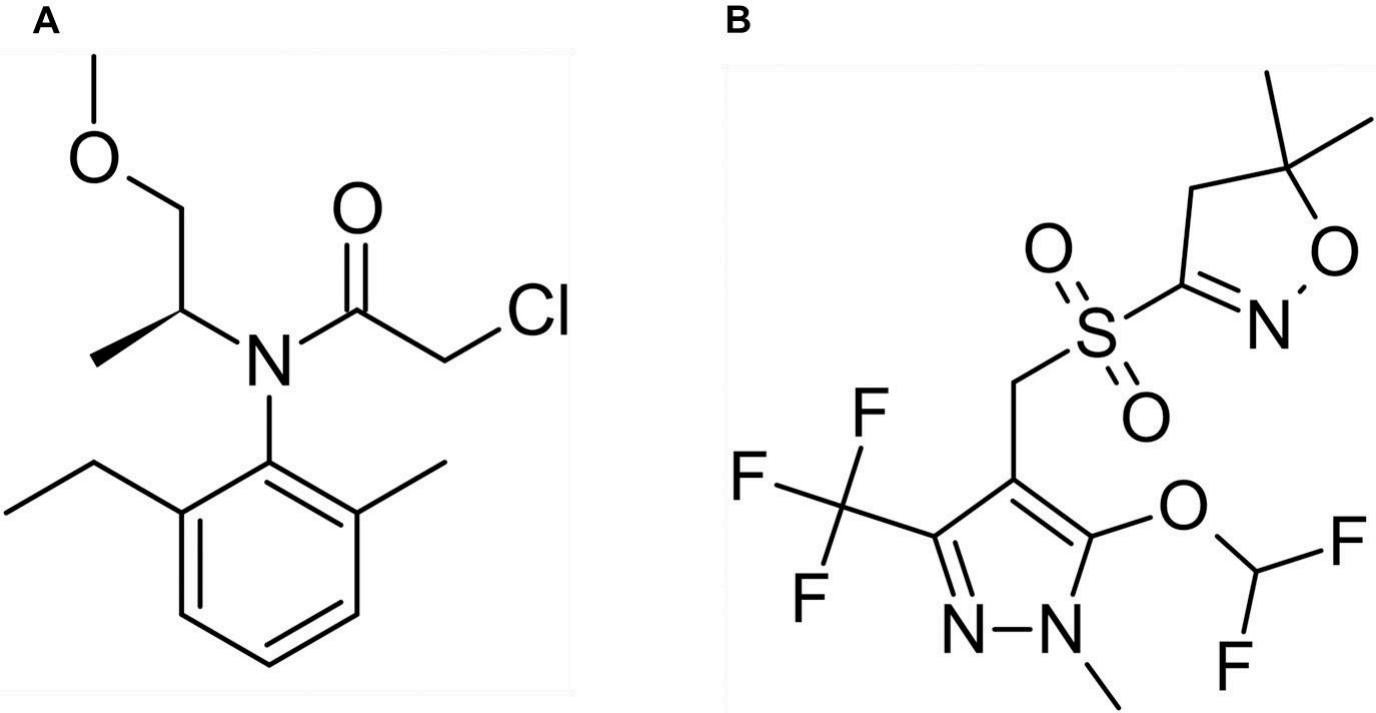
Preemergence soil-applied herbicides, *S*-metolachlor (A) and pyroxasulfone (B).

*S*-metolachlor (2-chloro-*N*-(2-ethyl-6-methylphenyl)-*N*-[(1*S*)-2-methoxy-1-methylethyl]acetamide) and pyroxasulfone (3-{[5-(difluoromethoxy)-1-methyl-3-(trifluoromethyl)-1H-pyrazol-4-yl]methanesulfonyl}-5,5-dimethyl-4,5-dihydro-1,2-oxazole) (Fig 1) are VLCFA-inhibiting herbicides (Group 15) belonging to the chloroacetamide and pyrazole chemical families, respectively [12,13]. *S*-metolachlor and pyroxasulfone control annual grass and broadleaf weeds PRE, including waterhemp [*Amaranthus tuberculatus* (Moq.) J.D. Sauer] [3,4,8,14] and other weeds in the Amaranthaceae. VLCFA-inhibiting herbicides inhibit the biosynthesis of nonsphingolipid VLCFAs (>18-carbon chain length) catalyzed by VLCFA elongase enzymes localized in the endoplasmic reticulum [12,15]. Inhibition of VLCFA biosynthesis depletes sensitive plants of cell membrane components and cuticle waxes [16–19]. Although pyroxasulfone and *S*-metolachlor share the same site-of-action (SoA) [18,19], these herbicides possess different chemistries, leading to differences in water solubility and vapor pressure [20–22]. In terms of weed control, these differing chemical properties can potentially produce varying levels of control of the same weed species [22].

Waterhemp is a highly competitive species capable of reducing maize and soybean yields by 36% and 43%, respectively [23,24]. Waterhemp is an annual dicot with C4 physiology and is a prolific seed producer [25]; in addition, seeds can remain dormant in the soil for multiple years [26]. Waterhemp also exhibits discontinuous germination throughout the growing season [27,28], which poses difficulty for control with PRE residual herbicides and might be mistaken as herbicide resistance. Waterhemp is dioecious and thus an obligate outcrosser [29,30], with male and female plants capable of passing herbicide resistance alleles (among other traits) to their offspring via pollen and seeds, respectively [31–35]. Obligate outcrossing contributes to increased genetic variability, and in turn, widespread distribution of herbicide resistance among waterhemp populations [36–38]. As an agronomically-important weed species, PRE herbicide-resistant waterhemp populations require rapid identification and early control [39].

Identification and quantification of weed resistance to PRE herbicides can be challenging because of inherent edaphic [40–42] and environmental factors. Edaphic factors such as soil structure, organic matter content, moisture, temperature, pH, and salinity affect the adsorption of herbicides to the soil, which then determines the bioavailability of the herbicide for weed control [43]. Moreover, herbicide application rate, active ingredient formulation, and amount of rainfall also affect control of many target weeds. In addition, PRE herbicides can display reduced efficacy from volatilization, UV light degradation on the soil surface, and microbial degradation if not properly incorporated into the soil [41,44]. Proper incorporation of Group 15 and many other soil-applied herbicides rely on accurate weather forecasts so applications can ideally be synchronized with a significant rainfall event (>2.5 cm) to ensure proper soil contact is made [14,43,45].

Due to increasing herbicide resistance cases in waterhemp populations [38] and the underlying challenges of soil-based and/or field testing, rapid and efficient diagnostic tests for early screening of herbicide resistance is needed. Soilless or hydroponic assays designed to screen plant species for herbicide sensitivity have been reported and reviewed [39,46–52]. However, these methods (1) typically require a climate-controlled growth facility or chamber, which are not readily available in every laboratory to accurately phenotype hundreds of plants, and/or (2) have not been used to identify and quantify dicot weed resistance to VLCFA-inhibiting herbicides. Pioneering soilless phenotyping assays were mainly developed for screening herbicide tolerance in crops [46–49]. For example, a soilless hydroponic system was developed for screening soybean cultivars after application of the soil-applied herbicide, metribuzin [46]. Another soilless assay used Petri dishes with media saturated in Murashige-Skoog salts to investigate root growth in sunflower (*Helianthus annuus* L.) after treatment with the acetolactate synthase (ALS)-inhibiting herbicide, imazapyr [48]. The “Resistance In-Season Quick” (RISQ) test is an agar-based, soilless assay for herbicide resistance screening in weeds [50]. The RISQ assay detected resistance to postemergence ALS- (Group 2) and acetyl-CoA carboxylase-inhibiting herbicides (Group 1) in weedy *Lolium* spp. and later was successfully adapted for identification of glyphosate resistance in *Lolium* populations, horseweed (*Erigeron canadensis*), goosegrass (*Eleusine indica*), and waterhemp under greenhouse conditions [51]. More recently, the RISQ assay was adapted for screening resistance of another weedy grass, *Alopecurus myosuroides*, to the PRE herbicides flufenacet (Group 15; VLCFA elongase inhibitor) and cinmethylin (Group 30; fatty-acid thioesterase inhibitor), which enabled identification of specific herbicide concentrations for control of sensitive weeds [52]. Agar-based resistance assays are adaptable for various herbicides and weed species; however, these methods require initial microwave and refrigeration of agar media [50] and a growth facility maintained at 20°C [52], which is not readily available across laboratories. As a result, additional or alternative rapid screening methods should be explored for dicot weeds.

Since VLCFA-inhibiting herbicides are often applied in combination with other active ingredients in a tank mixture, detection of resistance to VLCFA inhibitors alone can be challenging. Moreover, continuous use of VLCFA-inhibiting herbicides in tank mixtures poses an additional concern for managing rapidly evolving, multiple herbicide-resistant (MHR) weed populations [53]. For example, resistance to *S*-metolachlor, pyroxasulfone and other VLCFA-inhibiting herbicides was reported in two waterhemp populations previously characterized as resistant to inhibitors of 4-hydroxyphenylpyruvate dioxygenase (HPPD), photosystem II (PS II), protoporphyrinogen oxidase (PPO), ALS, as well as synthetic auxin herbicides [4,54]. Metabolic resistance to *S*-metolachlor was attributed to increased microsomal P450 activity, rapid formation of the Phase I metabolite, *O*-demethylated *S*-metolachlor, in MHR waterhemp but not in sensitive waterhemp or corn, as well as responses of these MHR populations to malathion (a plant P450 inhibitor) plus *S*-metolachlor [55,56]. Therefore, our goal is to identify and quantify resistance to the VLCFA-inhibiting herbicides, *S*-metolachlor and pyroxasulfone, using a novel growth assay designed to limit effects of edaphic factors. This method is henceforth called ‘PRIM’ (Preemergence Resistance Identification Method). Specifically, our research aims to: (i) develop a soilless dose-response assay for *S*-metolachlor and pyroxasulfone using exfoliated vermiculite in the greenhouse; (ii) conduct dose-response analyses of VLCFA inhibitor-sensitive and MHR waterhemp populations to *S*-metolachlor and pyroxasulfone; and (iii) determine potential interactions between *S*-metolachlor and malathion in these waterhemp populations.

## Materials and methods

### Plant materials

Two MHR and two VLCFA-inhibitor sensitive waterhemp populations were studied. The two MHR populations are: Stanford, Illinois Resistant (SIR) [4,59,60] and Champaign County, Illinois Resistant (CHR) [4,54]. SIR and CHR are resistant to VLCFA-inhibiting herbicides as well as herbicides from other SoA groups (inhibitors of HPPD, PPO, PSII and ALS as well as synthetic auxins) [4,10,57]. The two populations sensitive to VLCFA-inhibiting herbicides are: standard sensitive population (SEN) [55] and Adams County, Illinois Resistant (ACR population; ALS-inhibitor-, PPO-inhibitor- and atrazine-resistant but VLCFA-inhibitor sensitive) [58,59]. Both SEN and ACR were used to calculate resistant-to-sensitive ratios, or resistance indices (RI).

### Seed preparation

Seeds from each of the four waterhemp populations were stratified to break dormancy using a method previously described [60]. Stratified waterhemp seeds were pre-germinated on moistened filter paper sealed in a 100 cm^2^ Petri plate (Fisher Scientific, Hanover Park, IL 60133) with parafilm. Petri plates were then placed in a greenhouse with settings maintained at 28/22°C day/night under a 16 h photoperiod and incubated for 48–72 h or until most seedlings displayed a radicle, confirming that only live seedlings were subjected to herbicide treatments.

### Herbicides for dose-response analysis in vermiculite growing media (PRIM)

**Fig 2:**
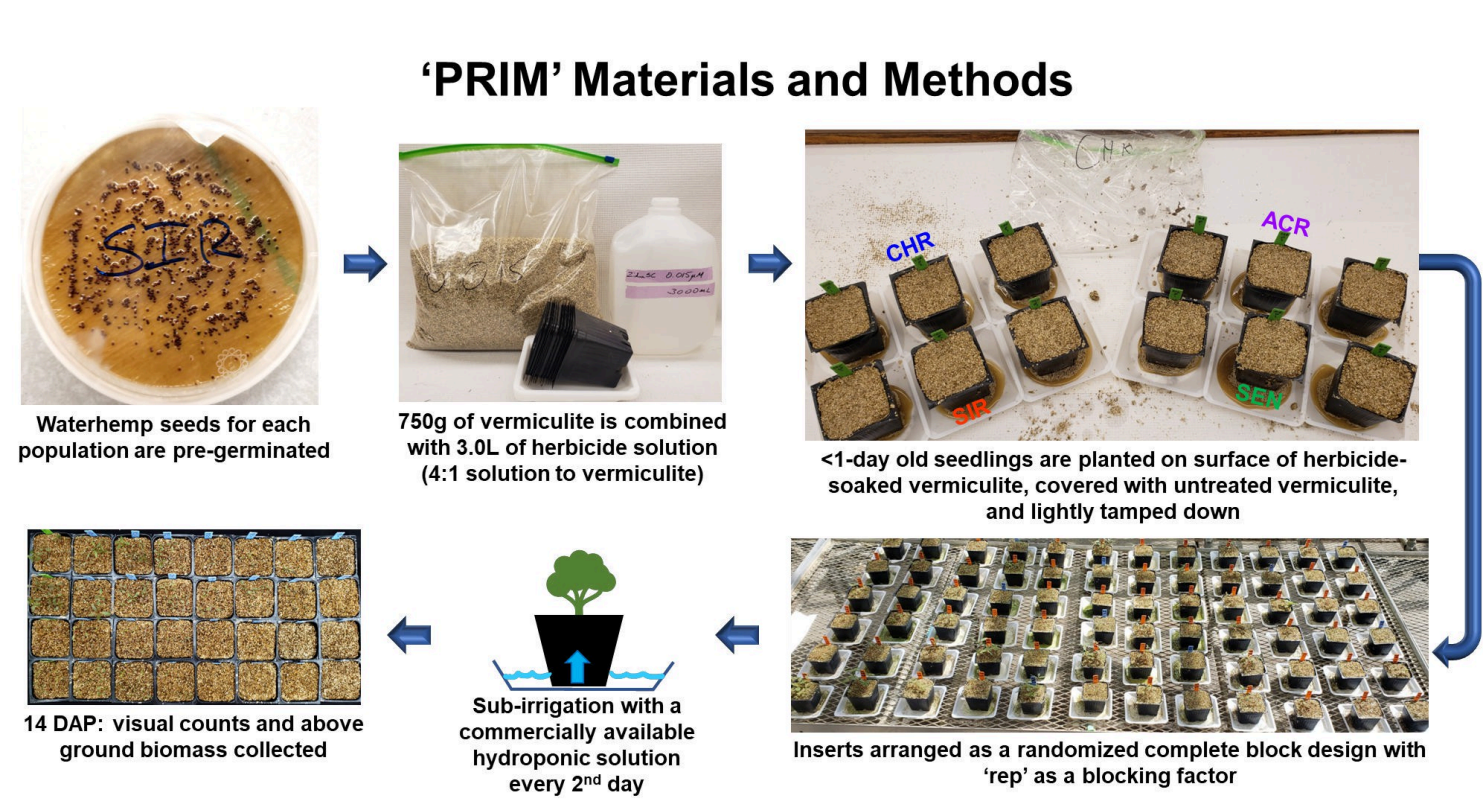
Preemergence resistance identification method (PRIM) uses basic greenhouse supplies, access to greenhouse space and small amounts of chemicals.

A soilless assay using exfoliated vermiculite was developed to determine the response of waterhemp populations to varying concentrations of *S*-metolachlor and pyroxasulfone (Fig 2). Exfoliated vermiculite consists of soil fractions expanded into elongated and layered particles via high temperature [61]. In our study, dry, medium-textured, exfoliated vermiculite (Thermo-O-Rock East Inc., New Eagle, PA 15067 USA) was used as the growing medium (combined with nutrient solution) due to its sterility, chemically neutrality, water insolubility, lack of electrical conductivity, and capacity to imbibe liquids during an extended time period [61]. These properties of exfoliated vermiculite prevent microbial infestation and binding of herbicides to the medium (such as soil), as well as allow for plant uptake of herbicides in solution.

Vermiculite was weighed and placed into sealable 9.5-L plastic bags. Each bag containing 750 g of vermiculite was combined with 3L of herbicide solution and sealed, representing a 4:1 ratio of vermiculite-to-herbicide solution (Fig 2). Seven concentrations of formulated *S*-metolachlor (Dual Magnum®, Syngenta Crop Protection, Greensboro, NC 27419 USA) and eight concentrations of formulated pyroxasulfone (Zidua®, BASF Corporation Agricultural Products, Research Triangle Park, NC 27709 USA) were used for the experiment. Herbicide concentrations were spaced by a factor of 3.16 and diluted in reverse-osmosis water. *S*-metolachlor concentrations were 0.015, 0.05, 0.15, 0.5, 1.5, 5 and 15 μM, whereas pyroxasulfone concentrations were 0.0005, 0.0015, 0.005, 0.15, 0.05, 0.15, 0.5 and 1.5 μM. Sealed bags were gently rolled and massaged by hand to ensure a homogenous mixture of vermiculite and herbicide. This herbicide-saturated vermiculite was then randomly placed into twelve-509 cm^3^ cell pack inserts (31801 Deep Insert, BFG Supply Company, Janesville, WI 53546 USA) (three inserts per population) and placed onto a 10 cm × 10 cm weigh boat. Herbicide solution was collected in the weigh boat and then poured onto the vermiculite surface to further incorporate the solution into the soilless medium. Pre-germinated waterhemp seeds from each of the previously described populations (CHR, SIR, ACR and SEN) were planted on the vermiculite surface at a rate of 10 seeds per insert, covered with a layer of untreated vermiculite, and lightly patted down by hand to ensure the added vermiculite absorbed the herbicide solution and provided optimum vermiculite-to-seed contact. Inserts were then arranged in the greenhouse in a randomized complete block design using ‘rep’ as a blocking factor.

Greenhouse conditions were maintained at 28/22°C day/night under a 16 h photoperiod. Supplemental sunlight was provided using mercury halide lamps providing 800 μmol m^-2^ s^-1^ photon flux to the vermiculite surface. Once placed in the greenhouse, each insert was sub-irrigated with 150 mL one-third strength commercial hydroponic fertilizer solution (Peters Hydroponic Special 5-11-26; ICL Specialty Fertilizers, Summerville, SC, USA) and supplemented with 0.15 g L^−1^ Ca(NO_3_)_2_ without chemical treatments the following day and then every second day for the duration of the experiment. Total number of surviving plants per pot was recorded 14 days after treatment (DAT), then plants were cut at the vermiculite surface, bagged, and placed in a 65°C oven. After approximately 48 h, dry weights were recorded and compared to their respective untreated controls. The experiment was performed twice independently (separated in time) and data were pooled for further statistical analysis.

### *S*-metolachlor and malathion interaction study

Preparation of herbicide-malathion-vermiculite media for PRIM was carried out the same as for *S*-metolachlor and pyroxasulfone alone, except the addition of malathion (Spectracide® Malathion Insect Spray Concentrate, Spectrum Group, Division of United Industries Corporation, St. Louis, MO 63114, USA) and only studying one VLCFA-inhibitor-resistant population (CHR) and VLCFA-inhibitor-sensitive population (ACR) due to limited seed and space availability. *S*-metolachlor concentrations ranging from 0.015 to 15 μM, with and without 2.0 μM malathion, were used for the experiment. This malathion concentration (2.0 μM) did not significantly affect seedling survival or growth in preliminary experiments (S1 Fig) and hence was deemed appropriate for the inhibitor assay. Control plants consisting of five biological replicates (ten seedlings per replicate) of the CHR and ACR populations were treated with the same nutrient solution described previously. After applying treatments, pots were moved to the greenhouse and arranged in a completely randomized design for the duration of the experiment. At 14 DAT, the total number of surviving plants per pot was recorded, then plants were cut at the vermiculite surface, bagged, and placed in a 65°C oven. After approximately 48 h, dry weights were recorded and compared to their respective untreated controls. The experiment was performed twice independently and data were pooled for further statistical analysis.

### Statistical analysis

Dose-response experiments with *S*-metolachlor and pyroxasulfone alone were performed separately and each was conducted twice. Dose-response experiments for the *S*-metolachlor-malathion interaction study were performed simultaneously and conducted twice. Biomass and survival data per study were pooled since the O’Brien test for homogeneity of variance for repeated experiments was not significant. Survival and biomass data were analyzed using a three-parameter logistic regression model $y=\frac{d}{1+\exp\left\{b[\log\left(x\right)-\log\left(e)\right]\right\}}$ [62], where *d* is the upper limit, *b* is the slope of the curve, and *e* is the 50% reduction in seedling survival (LD_50_) or 50% reduction in seedling aboveground dry biomass (GR_50_), in the ‘Analysis of Dose-Response Curves’, or *drc* package, in R (Version 3.4.3) and RStudio (Version 1.2.1335) [63]. Lethal dose estimates of 50% (LD_50_) and growth reduction estimates of 50% (GR_50_) for each population were obtained from each analysis, respectively. Finally, RI values for MHR waterhemp populations (CHR and SIR) for *S*-metolachlor and pyroxasulfone dose-response experiments were calculated using the equation: RI = (LD_50_/GR_50-resistant_) / (LD_50_/GR_50-sensitive_), where LD_50_/GR_50_-_sensitive_, is the concentration of herbicide causing 50% reduction in phenotypic response in the SEN and ACR populations relative to untreated controls at 14 DAT. The RI for the *S*-metolachlor plus malathion interaction study was calculated similarly as the ratio between LD_50_ or GR_50_ value of CHR relative to the LD_50_ or GR_50_ of ACR.

## Results

### Response of waterhemp populations to VLCFA-inhibiting herbicides

**Fig 3:**
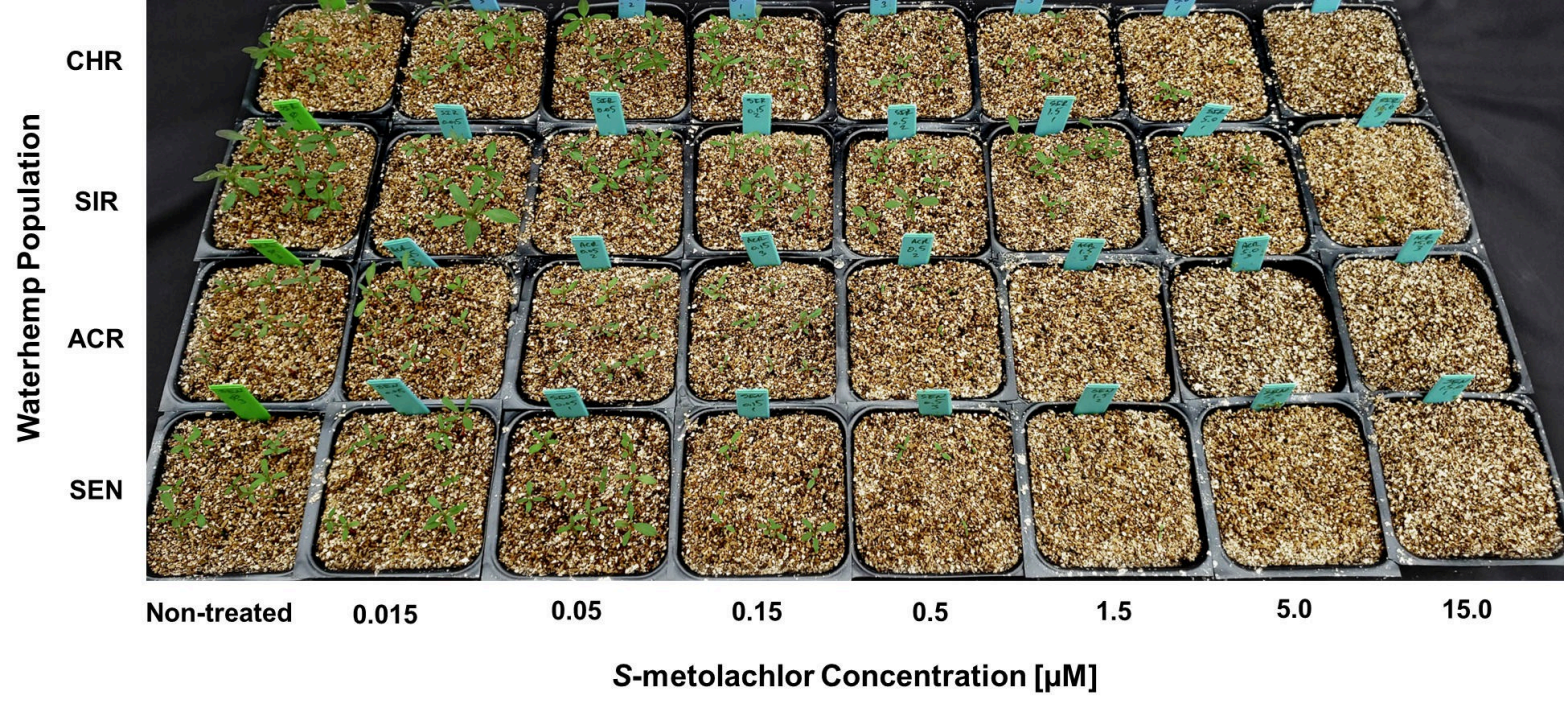
Responses of four waterhemp (*Amaranthus tuberculatus*) populations to *S*-metolachlor applied preemergence. *S*-metolachlor concentrations ranged from 0.015 to 15 μM. Seedlings are shown at 14 days after treatment (DAT). Non-treated inserts appear on the left for each population and treated inserts are arranged from left-to-right with increasing herbicide concentrations. Herbicide treatments using the PRIM assay are described in Materials and methods.

**Fig 4:**
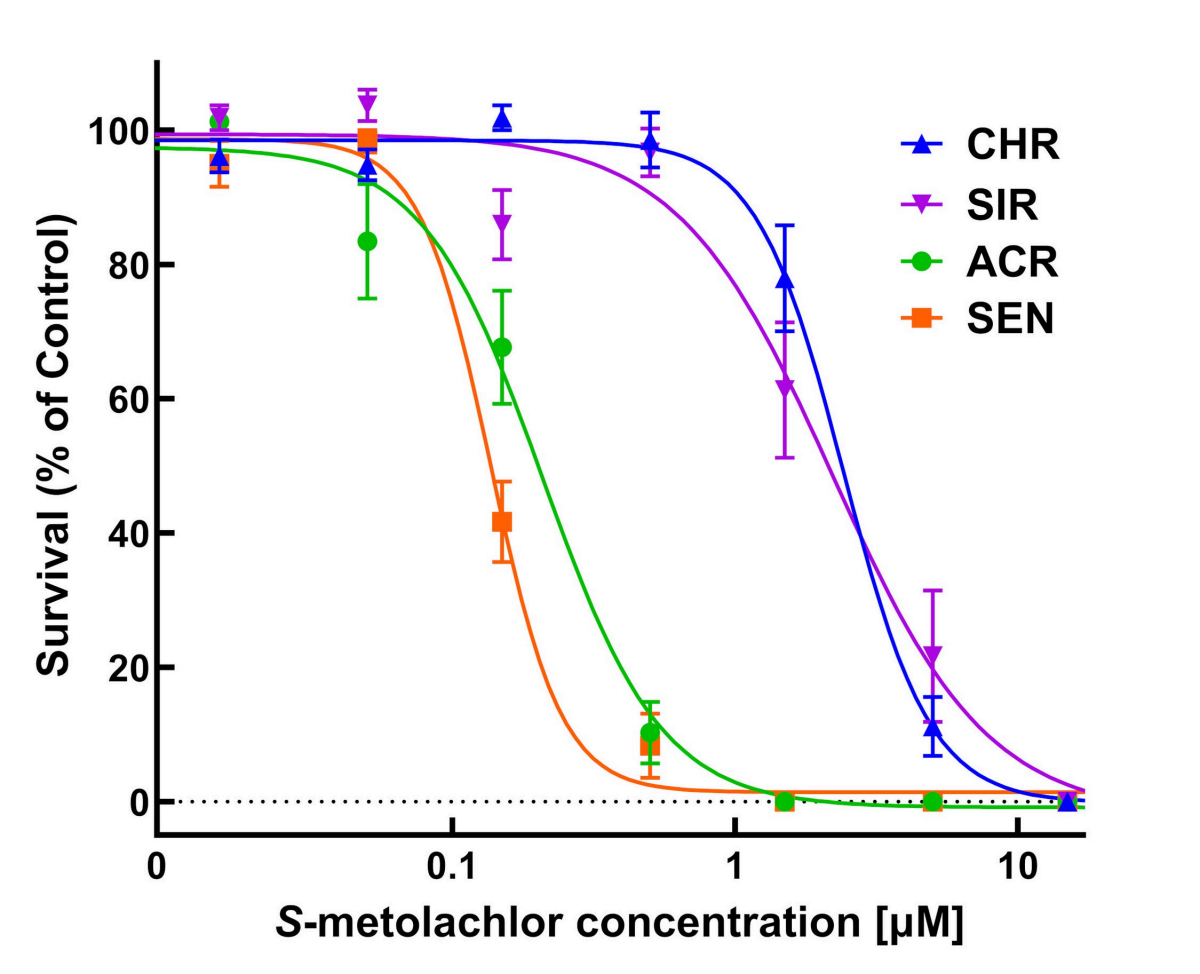
Quantitative survival analysis of CHR, SIR, ACR, and SEN populations in response to *S*-metolachlor. Dose-response analysis of four waterhemp (*Amaranthus tuberculatus*) populations in herbicide-treated vermiculite using PRIM. Data were collected 14 days after treatment (DAT) by counting the number of surviving plants. Results are presented as a percentage of the untreated control for each population. Dose-response curves were fitted using the equation $y=\frac{d}{1+\exp\left\{b[\log\left(x\right)-\log\left(LD50)\right]\right\}}$ and each symbol’s error bar represents ±SE. CHR, solid line and solid triangle; SIR, solid line and solid inverted triangle; ACR, solid line and solid circle; SEN, solid line and solid square.

**Fig 5:**
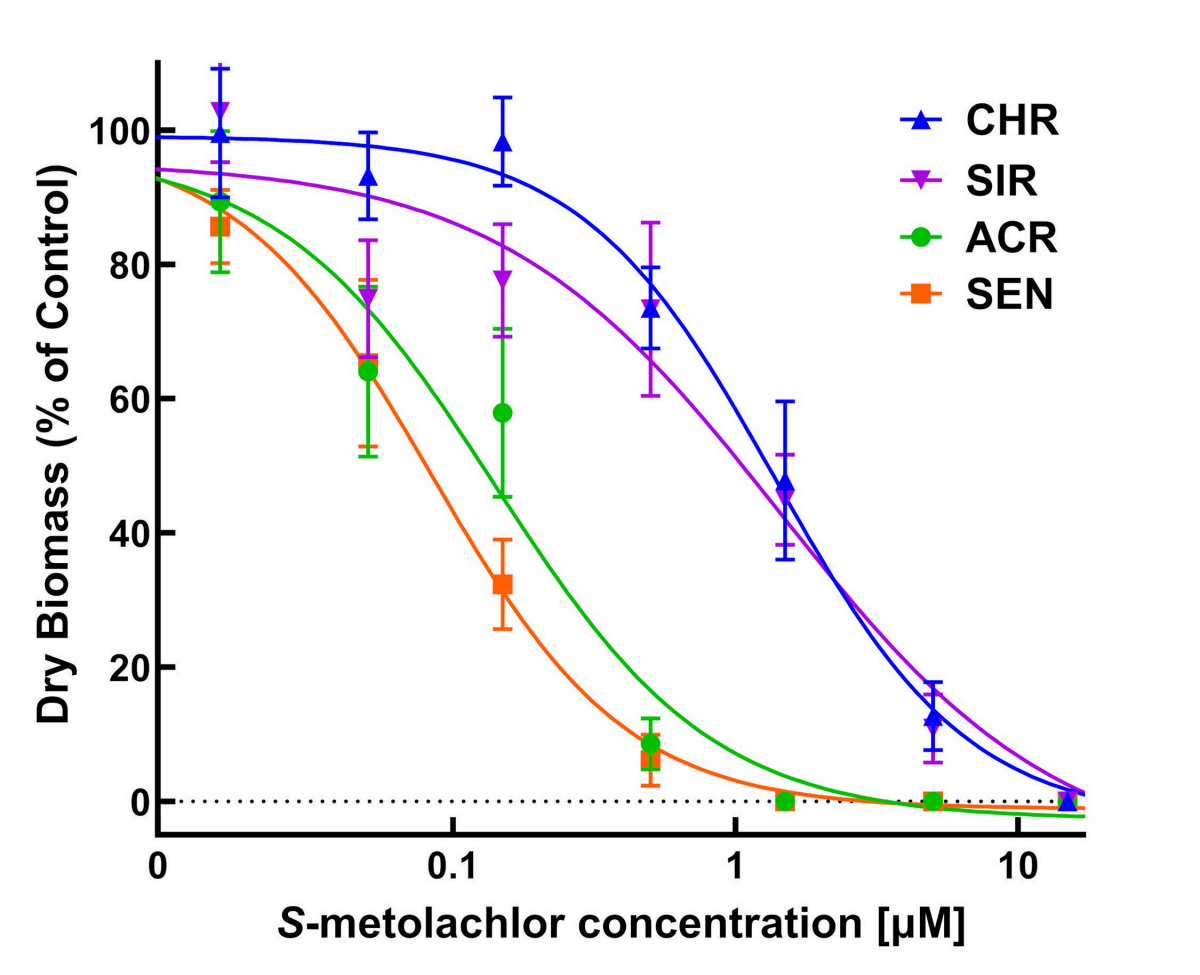
Quantitative growth reduction analysis of CHR, SIR, ACR, and SEN populations in response to *S*-metolachlor. Dose-response analysis of four waterhemp (*Amaranthus tuberculatus*) populations in herbicide-treated vermiculite using PRIM. Plants were harvested 14 days after treatment (DAT), dried in an oven, and aboveground dry biomass of surviving plants is expressed as a percentage of the untreated control. Dose-response curves were fitted using the equation $y=\frac{d-c}{1+\exp\left\{b[\log\left(x\right)-\log\left(LD50)\right]\right\}}$ and each symbol’s error bar represents ±SE. CHR, discontinuous line and solid circle; SIR, discontinuous line and solid triangle; ACR, solid line and solid square; SEN, discontinuous line and solid diamond.

Dose-response experiments with *S*-metolachlor using PRIM demonstrated significant differences between the two MHR waterhemp populations (CHR and SIR) and two VLCFA-sensitive populations (SEN and ACR) (Table 1; Figs 3–5). In response to *S*-metolachlor, RI values calculated using LD_50_ values for CHR and SIR were 17.2 and 15.2, respectively, compared to SEN, or 11.5 and 10.1 compared to ACR (Table 1). RIs calculated using GR_50_ values for CHR and SIR were 14.5 and 14.4, respectively, compared to SEN, or 9.7 and 9.6 compared to ACR (Table 1). In general, these RI values for CHR and SIR are relatively similar when measuring different dose-response parameters relative to SEN or ACR.

**Table 1:** Mean lethal dose estimates of 50% (LD_50_) and growth reduction estimates of 50% (GR_50_) in waterhemp (*Amaranthus tuberculatus*) using *S*-metolachlor.

Population	*S*-metolachlor responses			
	LD_50_ (μM)^a^	GR_50_ (μM)^a^	RI (LD_50_)^b^	RI (GR_50_)^b^
**CHR**	2.41 (±0.21)	1.16 (±0.23)	17.2, 11.5	14.5, 9.7
**SIR**	2.13 (±0.21)	1.15 (±0.33)	15.2, 10.1	14.4, 9.6
**ACR**	0.21 (±0.02)	0.12 (±0.03)	-	-
**SEN**	0.14 (±0.01)	0.08 (±0.02)	-	-

^a^ Estimated values are expressed as *S*-metolachlor concentrations (μM) followed by standard errors of the mean in parentheses.

^b^ Resistance indices (RI) were calculated based on the sensitive populations, SEN and ACR, respectively.

**Fig 6:**
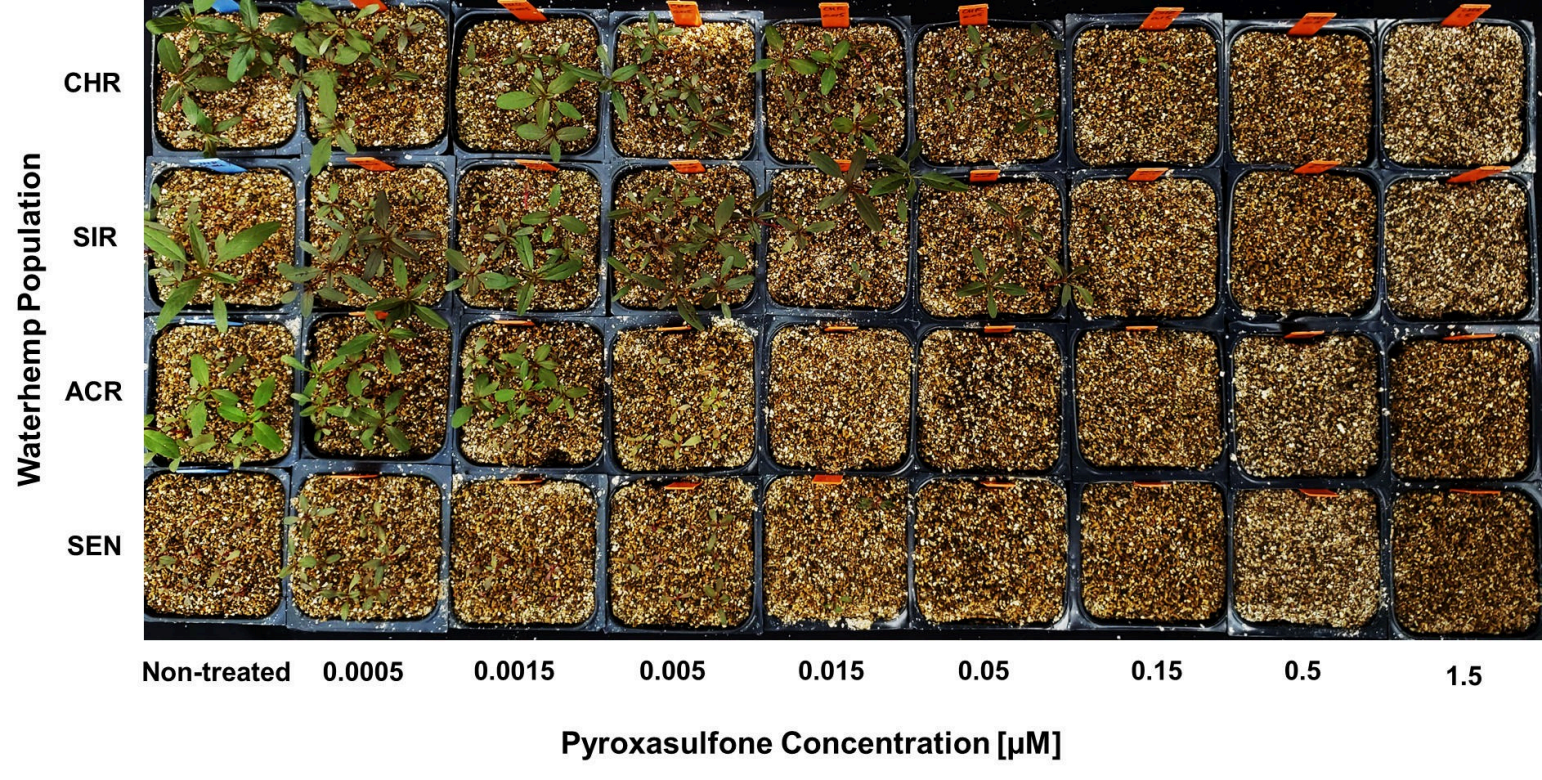
Responses of four waterhemp (*Amaranthus tuberculatus*) populations to pyroxasulfone applied preemergence. Pyroxasulfone concentrations ranged from 0.0005 to 1.5 μM. Seedlings are shown at 14 days after treatment (DAT). Non-treated inserts appear on the left for each population and treated inserts are arranged from left-to-right with increasing herbicide concentrations. Herbicide treatments are described in Materials and Methods.

**Fig 7:**
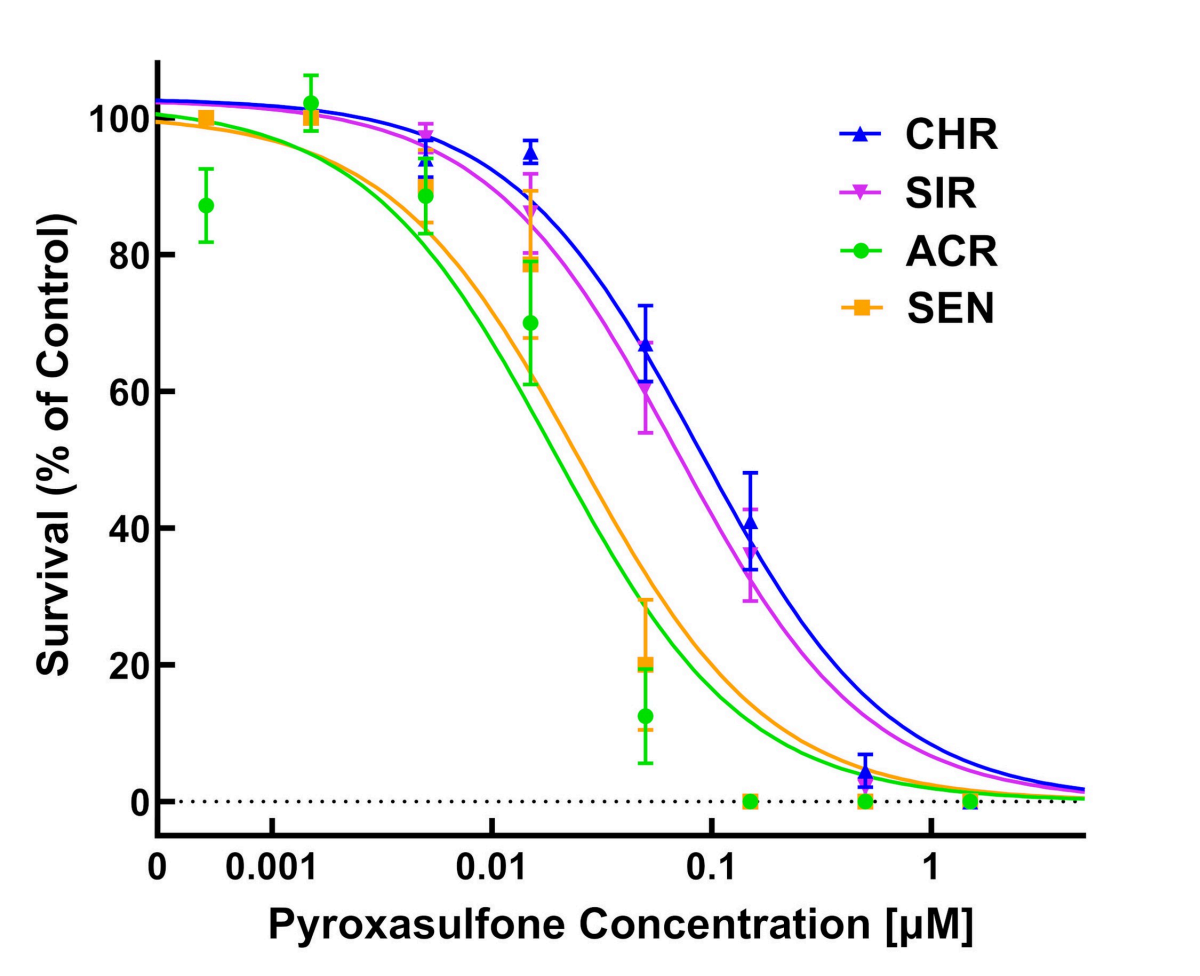
Quantitative survival analysis of CHR, SIR, ACR, and SEN populations in response to pyroxasulfone. Dose-response analysis of four waterhemp (*Amaranthus tuberculatus*) populations in herbicide-treated vermiculite using PRIM. Data were collected 14 days after treatment (DAT) by counting the number of surviving plants. Results are presented as a percentage of the untreated control for each population. Dose-response curves were fitted using the equation $y=\frac{d}{1+\exp\left\{b[\log\left(x\right)-\log\left(LD50)\right]\right\}}$ and each symbol’s error bar represents ±SE. CHR, discontinuous line and solid circle; SIR, discontinuous line and solid triangle; ACR, solid line and solid square; SEN, discontinuous line and solid diamond.

**Fig 8:**
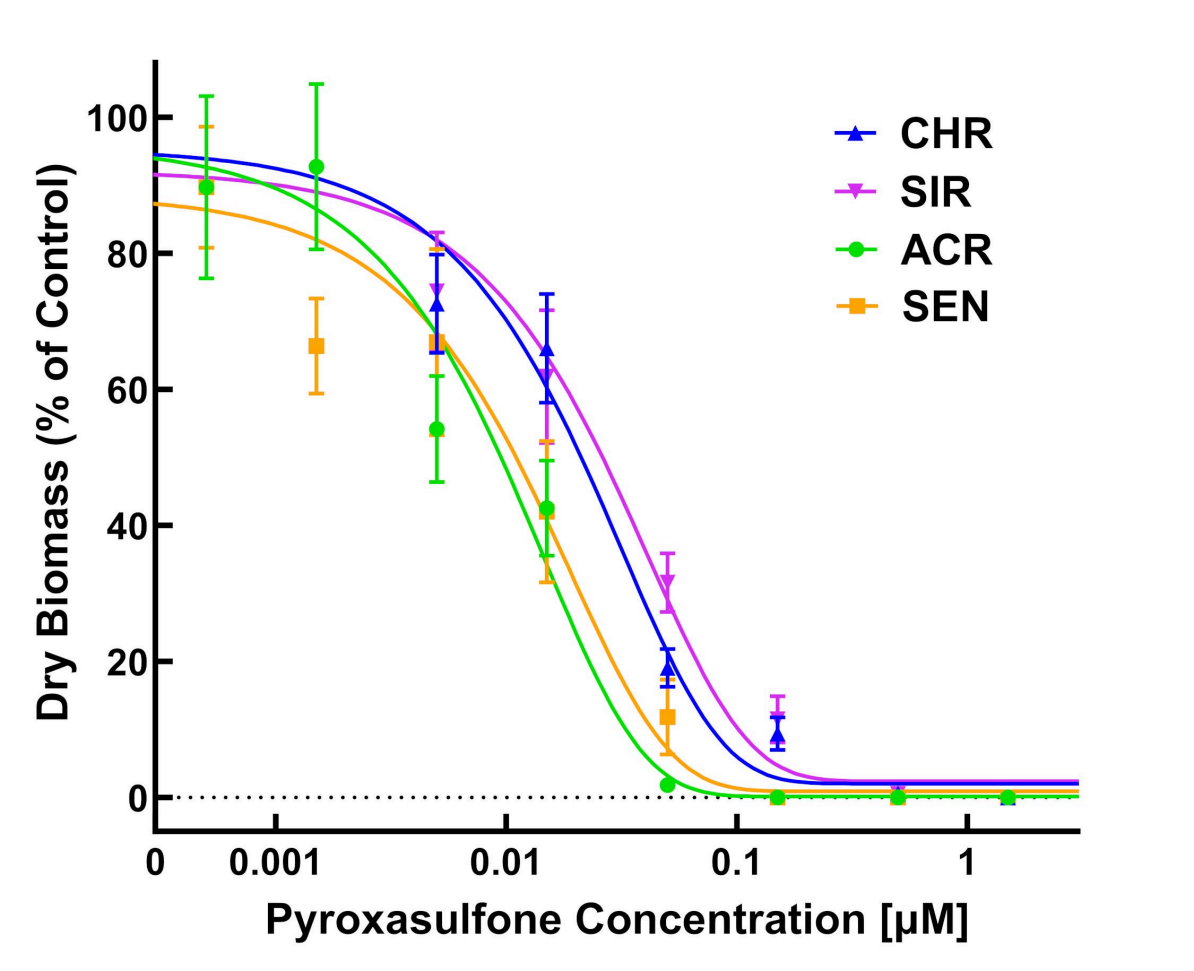
Quantitative growth reduction analysis of CHR, SIR, ACR, and SEN populations populations in response to pyroxasulfone. Dose-response analysis of four waterhemp (*Amaranthus tuberculatus*) populations in herbicide-treated vermiculite using PRIM. Plants were harvested 14 days after treatment (DAT), dried in an oven, and aboveground dry biomass of surviving plants is expressed as a percentage of the untreated control. Dose-response curves were fitted using the equation $y=\frac{d}{1+\exp\left\{b[\log\left(x\right)-\log\left(GR50)\right]\right\}}$ and each symbol’s error bar represents ±SE. CHR, discontinuous line and solid circle; SIR, discontinuous line and solid triangle; ACR, solid line and solid square; SEN, discontinuous line and solid diamond.

Dose-response experiments with pyroxasulfone using PRIM also showed significant differences between the two MHR populations (CHR and SIR) and two VLCFA-inhibitor sensitive populations (SEN and ACR) (Table 2; Figs 6–8). RIs calculated using LD_50_ values for CHR and SIR were 3.8 and 3.1, respectively, compared to SEN, or 4.8 and 3.8 compared to ACR (Table 2). RIs calculated using GR_50_ values for CHR and SIR were 4.8 and 5.6, respectively, compared to both SEN and ACR (Table 2). The RI values calculated for pyroxasulfone (Table 2) are approximately 2- to 4-fold lower than RIs calculated for *S*-metolachlor using the same parameters (Table 1). This finding is in accord with a higher level of resistance to *S*-metolachlor than pyroxasuflone (and other VLCFA-inhibiting herbicides) determined in soil-based greenhouse and field studies with SIR and CHR [4,64].

**Table 2:** Mean lethal dose estimates of 50% (LD_50_) and growth reduction estimates of 50% (GR_50_) in waterhemp (*Amaranthus tuberculatus*) using pyroxasulfone.

Population	Pyroxasulfone responses			
	LD_50_ (μM)^a^	GR_50_ (μM)^a^	RI (LD_50_)^b^	RI (GR_50_)^b^
**CHR**	0.10 (±0.01)	0.024 (±2.7x10^-3^)	3.8, 4.8	4.8, 4.8
**SIR**	0.08 (±0.009)	0.028 (±3.3x10^-3^)	3.1, 3.8	5.6, 5.6
**ACR**	0.021 (±0.002)	0.005 (±8.5x10^-4^)	-	-
**SEN**	0.026 (±0.003)	0.005 (±1.1x10^-3^)	-	-

^a^values are expressed as the *S*-metolachlor concentrations (μM) followed by standard errors of the mean in parentheses.

^b^ Resistance indices (RI) were calculated based on the sensitive populations, SEN and ACR, respectively.

### *S*-metolachlor-malathion interaction study

**Fig 9:**
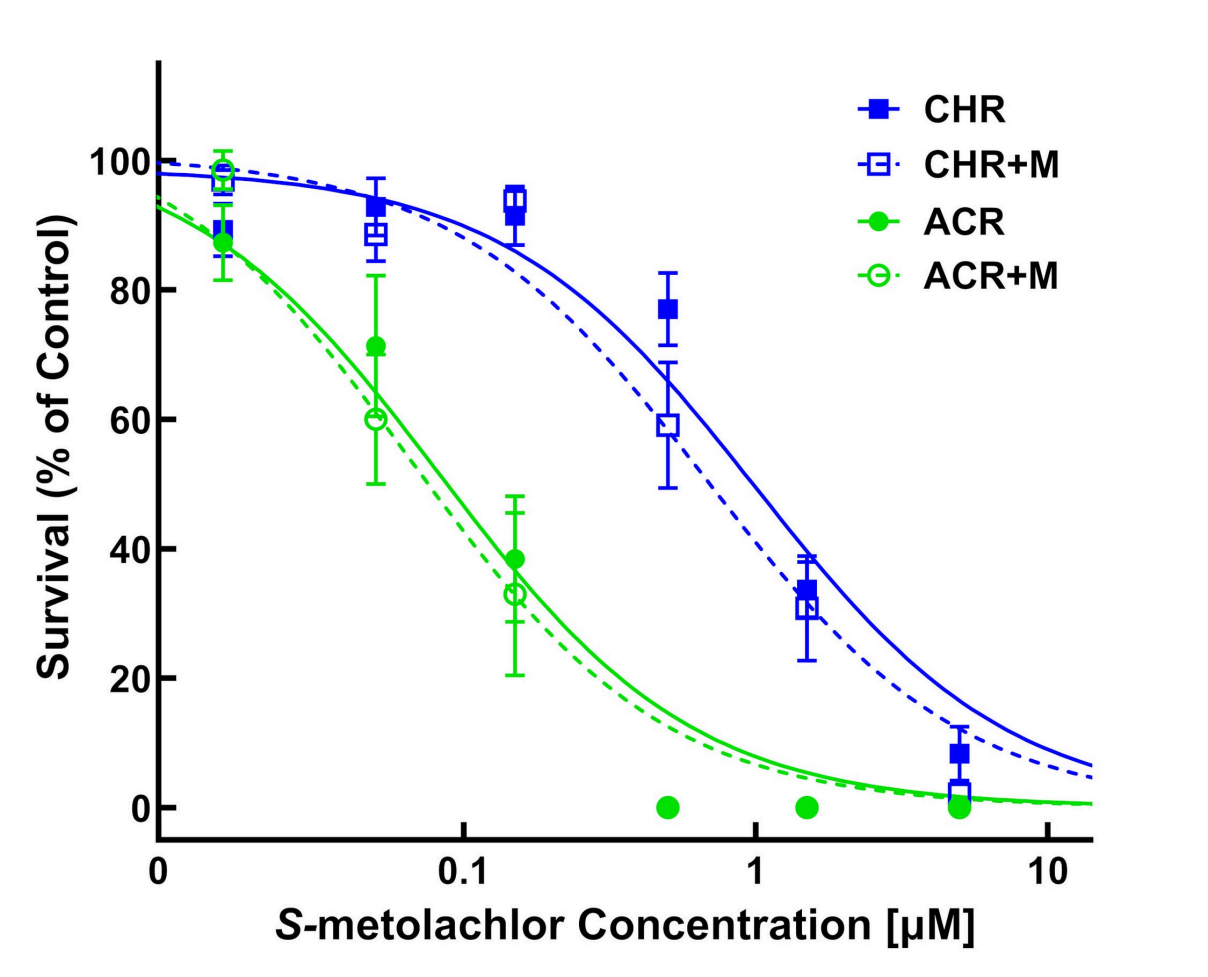
Quantitative survival analysis of CHR and ACR populations in response to *S*-metolachlor versus *S*-metolachlor plus malathion. Dose-response analysis of two waterhemp (*Amaranthus tuberculatus*) populations in *S*-metolachlor-treated and *S*-metolachlor plus malathion-treated vermiculite using PRIM. Data were collected 14 days after treatment (DAT) by counting the number of surviving plants. Results are presented as a percentage of the untreated control for each population. Dose-response curves were fitted using the equation $y=\frac{d}{1+\exp\left\{b[\log\left(x\right)-\log\left(LD50)\right]\right\}}$ and each symbol’s error bar represents ±SE. CHR, solid line and solid square; CHR+M, discontinuous line and hollow square; ACR, solid line and solid circle; ACR+M, discontinuous line and hollow circle. +M, treatment includes 2 μM malathion.

**Fig 10:**
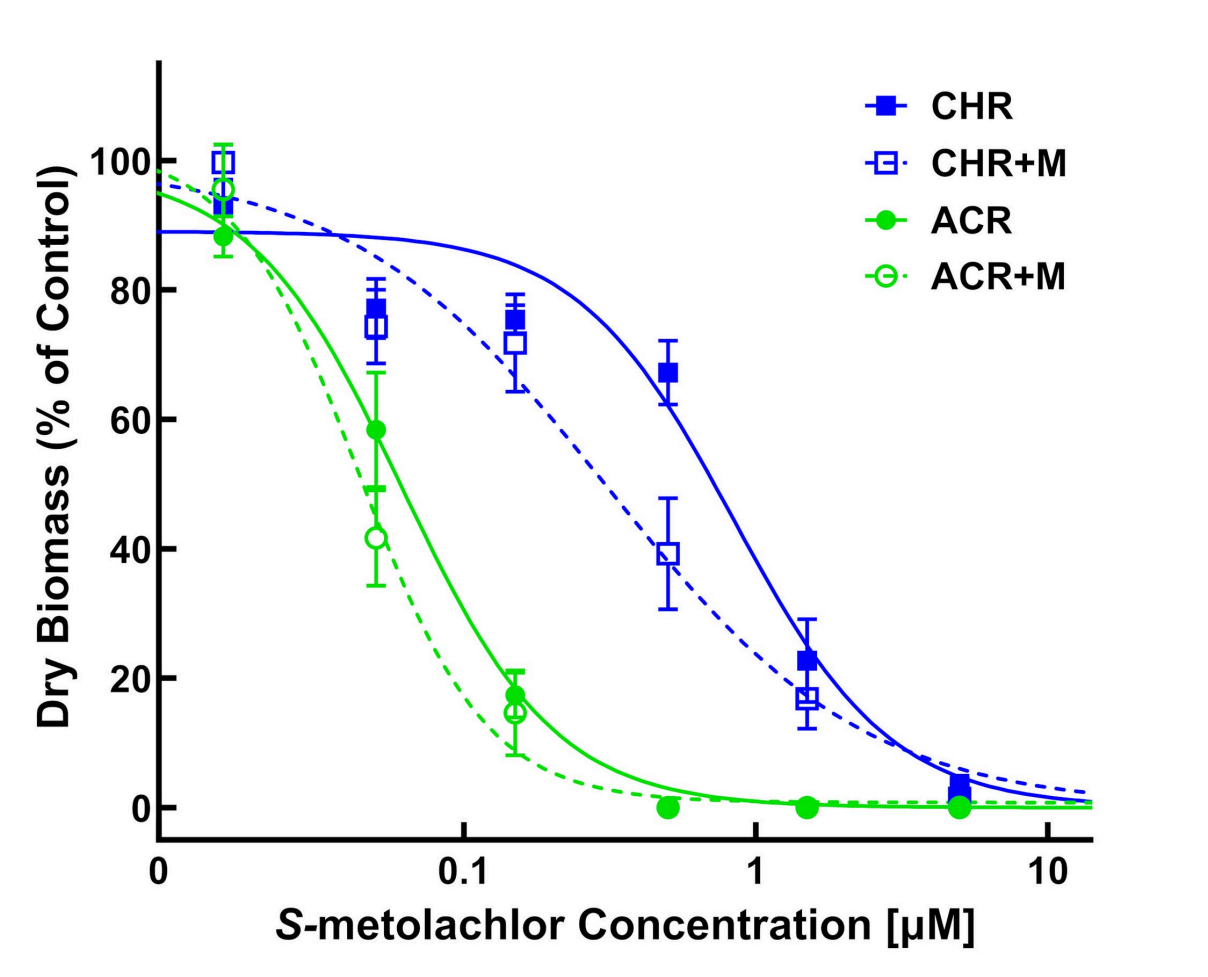
Quantitative growth reduction analysis of CHR and ACR populations in response to *S*-metolachlor versus *S*-metolachlor plus malathion. Dose-response analysis of two waterhemp (*Amaranthus tuberculatus*) populations in *S*-metolachlor-treated and *S*-metolachlor plus malathion-treated vermiculite using PRIM. Plants were harvested 14 days after treatment (DAT), dried in an oven, and aboveground dry biomass of surviving plants is expressed as a percentage of the untreated control. Dose-response curves were fitted using the equation $y=\frac{d}{1+\exp\left\{b[\log\left(x\right)-\log\left(GR50)\right]\right\}}$ and each symbol’s error bar represents ±SE. CHR, solid line and solid square; CHR+M, discontinuous line and hollow square; ACR, solid line and solid circle; ACR+M, discontinuous line and hollow circle. +M, treatment includes 2 μM malathion.

Addition of the plant P450 inhibitor, malathion, with *S*-metolachlor showed a trend of increased sensitivity (survival and growth parameters) in MHR waterhemp (CHR) and the VLCFA inhibitor-sensitive population, ACR (Tables 3 and 4; Figs 9 and 10). Survival (LD_50_) values of the CHR and ACR populations to *S*-metolachlor were reduced approximately 30% by adding malathion and, as a result, RI values for CHR remained similar (Table 3). However, a greater effect of malathion plus *S*-metolachlor on seedling growth (GR_50_) of the CHR population compared to the ACR population led to a decrease in RI from 14.5 to 8.8, which is consistent with the greater degree of oxidative metabolism of *S*-metolachlor in MHR compared to sensitive waterhemp [55].

**Table 3:** Mean lethal dose estimates of 50% (LD_50_) of *S*-metolachlor or *S*-metolachlor plus malathion (2 μM) in waterhemp (*Amaranthus tuberculatus*).

Population	*S*-metolachlor		*S*-metolachlor + malathion	
	LD_50_ (μM)^a^	RI^b^	LD_50_ (μM)^a^	RI^b^
**CHR**	1.12 (±0.12)	11.2	0.76 (±0.09)	10.9
**ACR**	0.10 (±0.01)	-	0.07 (±0.01)	-

^a^ Estimated LD values are expressed as *S*-metolachlor concentrations (μM) followed by standard errors of the mean in parentheses.

^b^ Resistance indices (RI) were calculated based on the LD_50_ of CHR relative to ACR in the *S*-metolachlor only treatments or *S*-metolachlor plus malathion treatments.

**Table 4:** Mean growth reduction estimates of 50% (GR_50_) of *S*-metolachlor or *S*-metolachlor plus malathion (2 μM) in waterhemp (*Amaranthus tuberculatus*).

Population	*S*-metolachlor		*S*-metolachlor + malathion	
	GR_50_ (μM)^a^	RI^b^	GR_50_ (μM)^a^	RI^b^
**CHR**	0.87 (±0.12)	14.5	0.35 (±0.07)^**c**^	8.8
**ACR**	0.06 (±0.01)		0.04 (±0.004)	

^a^ Estimated GR values are expressed as *S*-metolachlor concentrations (μM) followed by standard errors of the mean in parentheses.

^b^ Resistance indices (RI) were calculated based on the GR_50_ of CHR relative to ACR in the *S*-metolachlor only treatments or *S*-metolachlor plus malathion treatments.

^c^ GR_50_ value is significantly lower than the GR_50_ in the *S*-metolachlor only treatment (p<0.05).

## Discussion

Identification of resistance in weeds is an important step in weed management [9], but screening for resistance to PRE herbicides can be a daunting task due to the presence of confounding edaphic factors [52]. For instance, to achieve consistent efficacy, higher rates of pyroxasulfone are required to reach the same level of weed control when applied to soils with high percent soil organic matter [65]. Development of soilless screening techniques for PRE herbicides addresses limitations in soil-based methods without compromising accuracy [39,52]. In our study, identification of resistance to *S*-metolachlor and pyroxasulfone was accomplished using detailed dose-response analysis [66] and the PRIM in the greenhouse. Despite differences in structures and properties, different concentrations of pyroxasulfone and *S*-metolachlor completely controlled VLCFA-inhibitor sensitive populations (Figs 3–8). Compared to other soilless assays developed for resistance screening, which require preparation of sterile agar medium [50,51] and a temperature-regulated plant growth facility or chamber [52], PRIM requires minimal preparation and has flexibility to adapt to any growth facility amenable for screening different species. This method also represents an improvement on prior soilless techniques, such as agar-based assays [39,52], in that PRIM is not limited by possible microbial contamination. Using PRIM, a researcher could screen hundreds of weed seedlings suspected of resistance to VLCFA-inhibiting or other PRE herbicides. For example, 3L of herbicide solution in this study effectively phenotyped *c*.*a*. 120–180 seedlings per suspected resistant population (Fig 2). PRIM also preserves intact roots for further phenotyping or genotyping since vermiculite can be easily washed away with water.

Dose-response assays using PRIM demonstrated 0.5 and 0.05 μM as effective concentrations for discriminating resistant from sensitive waterhemp for *S*-metolachlor and pyroxasulfone, respectively. This magnitude of difference in effective concentrations is consistent with field applications where pyroxasulfone is generally applied at *c*.*a*. one-tenth (150–250 g a.i. ha^-1^) the rate of *S*-metolachlor (1–2 kg a.i. ha^-1^), indicating consistent availability of herbicide uptake by seedlings [4]. Waterhemp plants developing aboveground biomass, as characterized by a fully expanded second true leaf at discriminating pyroxasulfone and *S*-metolachlor concentrations, are characterized as resistant using PRIM (Figs 3 and 6). A researcher might choose a higher and/or lower concentration in addition to the discriminating rate to account for temperature or light intensity changes based on the time of year [67]. Additional foliar-applied herbicides with different SoA that also possess PRE residual activity, such as ALS inhibitors, PPO inhibitors, HPPD inhibitors or atrazine, may also have potential with the PRIM assay to identify resistant weeds, but requires further research.

Incorporation of malathion into our dose-response experiment shows PRIM is adaptable for investigating resistance mechanisms in the presence of metabolic inhibitors. Increased sensitivity of CHR to *S*-metolachlor in the presence of malathion corroborates previous results from a laboratory-based metabolic resistance assay [55] and supports previous findings on P450 enzyme activity in metabolic detoxification of *S*-metolachlor via Phase I *O*-demethylation [56]. The PRIM assay might also be used to investigate responses of CHR and SIR populations to herbicides and chemical inhibitors of GST enzyme activities [68,69], which contribute to metabolic detoxification of multiple herbicides in *Alopecurus myosuroides* [67,69] and MHR waterhemp [58,59,70]. The ability to screen for and identify resistance, perform accurate dose-response analysis, and utilize metabolic inhibitors or synergists to understand non-target-site resistance make PRIM a robust phenotyping method.

## Conclusions

Results from the PRIM assay support previous findings in soil-based systems without requiring expensive research-grade cabinet sprayers, growth chambers, automated overhead misting systems, or rented land where MHR waterhemp populations are present. PRIM is thus a promising alternative to soil-based screening assays for studying PRE herbicide efficacy and resistance in waterhemp and possibly other broadleaf weeds, such as Palmer amaranth [71,72], as well as monocots. For researchers considering PRIM for resistance studies, we suggest collecting both LD_50_ or GR_50_ data to generate RI values, if space and time allow, to identify and precisely quantify resistance levels to PRE herbicides. However, if available resources allow for just one parameter to be recorded for calculating RIs, we suggest collecting LD_50_ data since the time required to obtain seedling dry weights can be omitted. With its potential of being scaled up or down to meet greenhouse experimental designs and capability to incorporate PRE herbicides and metabolic inhibitors, PRIM can be easily adapted by various researchers as an alternative to soil-based screening of herbicide resistance in weed populations.

## Supporting information

S1 Fig Comparison of aboveground dry biomass of three waterhemp (*Amaranthus tuberculatus*) populations 14 days after treatment with 2.0 μM malathion relative to an untreated control (CHK). Pooled data from two experimental runs were analyzed using PROC GLIMMIX 9.4. The designation ‘ns’ indicates treatment means are not significantly different at alpha = 0.05.
(DOCX)
