# Supplementary material for: Quantifying resistance to very-long-chain fatty acid-inhibiting herbicides in Amaranthus tuberculatus using a soilless assay
Source: PLoS One. 2023 Dec 22;18(12):e0295927. doi: 10.1371/journal.pone.0295927 (PMC10745185; doi:10.1371/journal.pone.0295927)
Supplement: S1 Fig — Pooled data from two experimental runs were analyzed using PROC GLIMMIX 9.4. The designation ‘ns’ indicates treatment means are not significantly different at alpha = 0.05. (DOCX) [file pone.0295927.s001.docx]

**PLoS ONE** – Quantifying resistance to very-long-chain fatty acid-inhibiting herbicides in *Amaranthus tuberculatus* using a soilless assay

**Supplementary Figure**


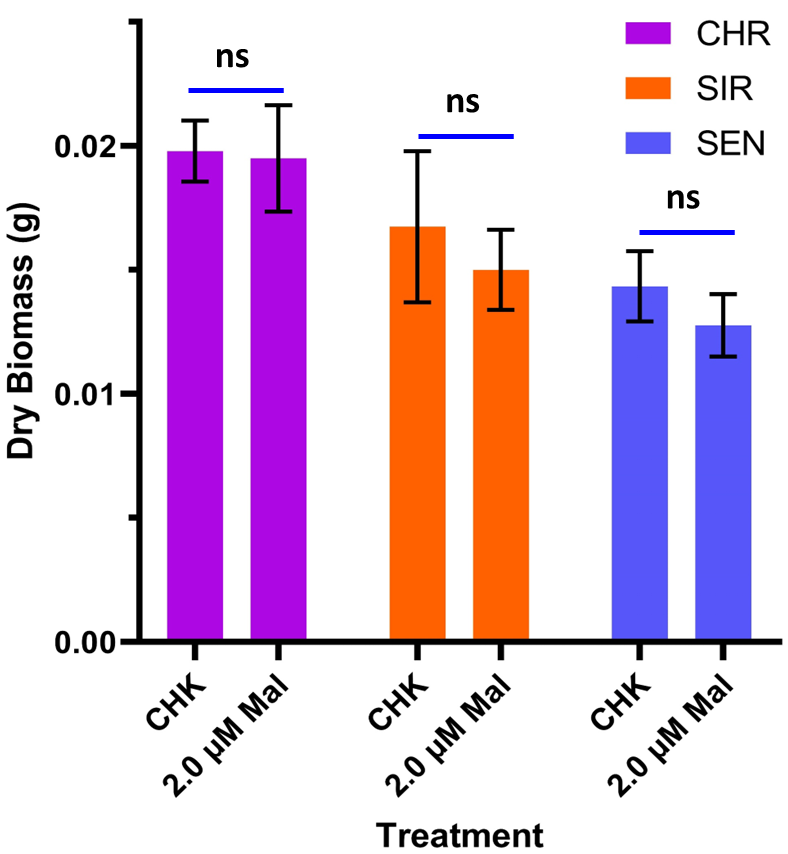


**S1 Fig.** Comparison of aboveground dry biomass of three waterhemp (*Amaranthus tuberculatus*) populations 14 days after treatment with 2.0 µM malathion relative to an untreated control (CHK). Pooled data from two experimental runs were analyzed using PROC GLIMMIX 9.4. The designation ‘ns’ indicates treatment means are not significantly different at alpha = 0.05.
